# Effects of intrahippocampal administration of the phosphatase inhibitor okadaic acid: Dual effects on memory formation

**DOI:** 10.1590/S1980-57642010DN40100004

**Published:** 2010

**Authors:** Monica R.M. Vianna, Adriana Coitinho, Luciana Izquierdo, Ivan Izquierdo

**Affiliations:** 1 Faculty of Biosciences, National Institute of Translational Medicine; 2 Memory Center, Brain Institute and National Institute of Translational Neuroscience, Pontifical Catholic University of Rio Grande do Sul, Porto Alegre RS, Brazil.

**Keywords:** hippocampus, PP1, PP2A, okadaic acid, short-term memory, long-term memory

## Abstract

Protein phosphorylation mediated by serine-threonine kinases in the hippocampus is crucial to the synaptic modifications believed to underlie memory formation. The role of phosphatases has been the focus of comparatively little study.

**Objectives:**

Here we evaluate the contribution of the serine-threonine protein phosphatases 1 and 2A (PP1, PP2A) on memory consolidation.

**Methods:**

We used immediate post-training bilateral hippocampal infusions of okadaic acid (OA, 0.01 and 10 pmol/side), a potent inhibitor of PP1 and PP2A, and measured short- [3 h] and long-term memory [24 h] (STM, LTM) of step-down inhibitory avoidance.

**Results:**

At the lower dose, OA inhibited both STM and LTM whereas at the higher dose it instead enhanced LTM. Pre-test infusion of these two doses of OA had no effect on retrieval.

**Conclusions:**

These two doses of OA are known to selectively inhibit PP1 and PP2A respectively. These findings point to the importance of these enzymes in memory formation and also suggest a deleterious influence of endogenous hippocampal PP2A on LTM formation.

## Abstract (pt)

A fosforilação de proteínas mediada por serina-treonina quinases no hipocampo é crucial para as modificações sinápticas que se acredita sejam necessárias para a formação de memórias. O papel das fosfatases tem sido comparativamente pouco estudado.

**Objetivos:**

Aqui avaliamos a contribuição das fosfatases serina-treonina 1 e 2 (PP1, PP2A) sobre a consolidação da memória.

**Métodos:**

Usamos infusões imediatamente após o treino de ácido okadaico (OA, 0.01 e 10 pmol/lado), um potente inibidor de PP1 e medimos memória de curta [3 h] e longa duração [24 h] (STM, LTM) de esquiva inibitória de evitar descer de uma plataforma.

**Resultados:**

Na dose menor, OA inibiu tanto STM como LTM. Na dose maior, produziu, em vez disso, uma melhora da LTM. A infusão pré-teste de qualquer uma das duas doses de OA não teve efeito sobre a evocação.

**Conclusões:**

Estas duas doses de OA são conhecidas por inibir seletivamente PP1 a PP2 respectivamente. Estes resultados apontam à importância das duas enzimas na formação de memória e sugerem, adicionalmente, uma influência deletérea da PP2A endógena sobre a formação de LTM.

Several serine-threonine protein kinases constitute signaling pathways whose activation is necessary for memory formation in the hippocampus.^1,2^ These include the calcium-calmodulin dependent kinase [CaMKII] that mediates GluR^1^ phosphorylation,^3,4^ the cAMP-dependent [PKA] and mitogen-activated kinases [MAPK] that mediate phosphorylation of the transcription factor CREB and other substrates,^5-7^ and the calcium-dependent protein kinase family (PKC) that also mediates the phosphorylation of many substrates, including presynaptic proteins involved in glutamate release.^8,9^ There is abundant cross-talk among all these kinase families.^7,10^ Their importance in memory suggests that serine-threonine phosphatases such as PP1, PP2A and calcineurin may also play a role.^11-14^ Indeed, inhibitors of PP1 and PP2A enhance NMDA currents in cultured hippocampal neurons,^15^ but antagonize the NMDA receptor-dependent inhibition of late-long term potentiation (LTP) caused by low frequency stimulation in hippocampal slices.^16^ Both hippocampal early NMDA currents and late LTP appear to be necessary for memory formation.^2,17,18^ Inhibitors of PP^1^, PP2A and calcineurin have been shown to have deleterious effects on various forms of memory.^11-13.19-21^ The best studied of these phosphatases is calcineurin, for which an allosteric model has been suggested in which, once bound to calmodulin, calcineurin competes with CaMKII for calcium.^14^ Calcineurin appears to govern both an intermediate phase of LTP between the so-called early and late phases,^22^ and the development of LTM for spatial and nonspatial tasks.^23^ The inducible and reversible genetic inhibition of calcineurin in mouse brain enhances learning, STM and LTM of hippocampus-dependent tasks and hippocampal LTP in a PKA-dependent manner.^24^

The influence of PP1 and PP2A on memory variables is less clear. Genetic inhibition of PP1 suppresses the deleterious effect of massed trials on learning, and prolongs memory duration.^25^ Suppression also decreases LTD and favors LTP in a frequency-dependent manner in the hippocampus.^26^ While these findings are important and point to a role of PP1 both in hippocampal plasticity and memory parameters, they are not illustrative, however, as to what specific phase of memory PP1 is involved in. No similar data are available for PP2A. Although some of the behavioral findings do suggest a different time course for the PP1 and PP2A influences on memory,^21^ it is not clear whether different forms of memory are affected by each. We have recently demonstrated a degree of independence of short-term memory lasting 3 h or less (STM) and long-term memory lasting one day or more (LTM), which are essentially parallel processes.^27,28^

Here we concentrate on the inhibition of hippocampal PP1 and PP2A by two widely differing dose concentrations of okadaic acid well known to selectively inhibit one or the other enzyme.^21,29,30^ We studied one-trial inhibitory avoidance in rats, a task equivalent to the one-trial peck avoidance task studied in the one-day-old chick by Bennett, Ng and their coworkers,^11,12.19,21^ which is also acquired in a few seconds and, in the rat, depends mainly on the hippocampus^2^. In addition, it is the task in which STM and LTM were shown to be functionally separate^27^ and where LTM was found to use the same molecular cascades as LTP.^2,18^

## Methods

Adult 3 month-old Wistar male rats (250-300 g) purchased from Fundação Estadual de Produção e Pesquisa em Saúde do Rio Grande do Sul, Porto Alegre were used. The animals were housed 5 to a cage and had free access to food and water under a 12/12 h light/dark cycle, with lights on at 7:00 AM. The temperature of the animal room was maintained at 22-24°C. To implant them with indwelling cannulae, rats were deeply anesthetized with thiopental (i.p., 30-50 mg/kg) and 27-gauge cannulae stereotaxically aimed at the CA1 region of the dorsal hippocampus, in accordance with coordinates (A ±4.3, L ±3.0, V 3.4) from the atlas of Paxinos and Watson.^31^ Animals were allowed to recover from surgery for 4 days before submitting them to any other procedure.

**Figure 1:**
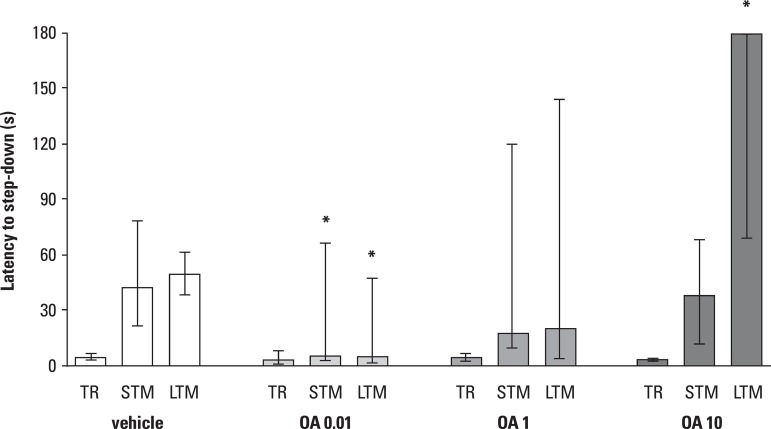
Effect of bilateral intrahippocampal infusions of OA at different concentrations (0.01, 1 and 10 pmol/side) immediately after step-down inhibitory avoidance training session. Control group received vehicle (20% dimethylsulfoxide in saline) in which OA was diluted. Columns indicate Medians (interquartile ranges) of step-down latencies in seconds, of training (TR) and STM and LTM tests for each group. Asterisks indicate significant statistical difference at p<0.05level on the Mann-Whitney U test, to the respective control groups in the respective session.

**Figure 2:**
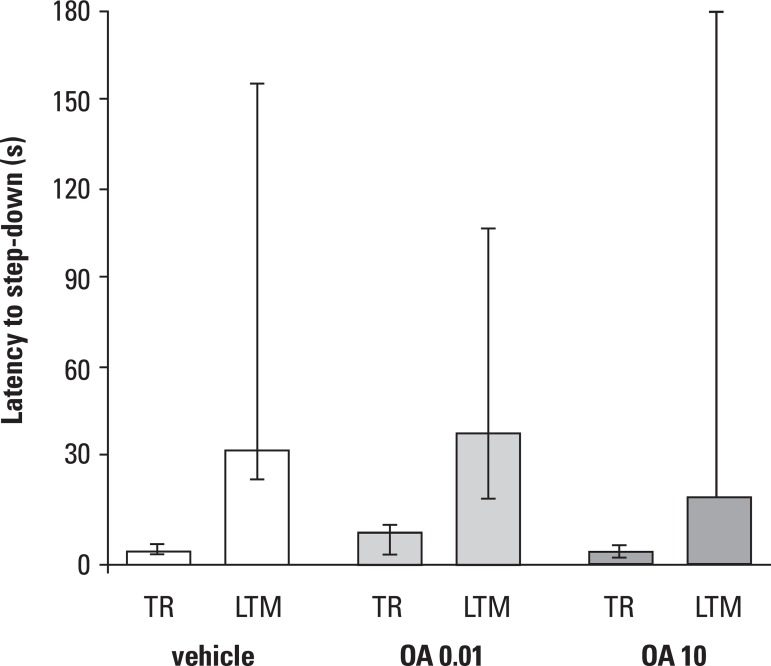
Effect of bilateral intrahippocampal infusions of OA on memory retrieval when given 15 min before test session. Control group received vehicle (20% dimethylsulfoxide in saline) in which Okadaic acid was diluted. Treated animals received Okadaic at 1 and 10 pmol/side. Columns indicate medians, and vertical lines indicate interquartile ranges of step-down latencies in seconds, of training (TR) and LTM test for each group. Asterisks indicate significant statistical difference at least at p<0.05level on the Mann-Whitney U test, to the respective control groups in the respective session.

At the time of drug delivery, 30-gauge infusion cannulae were tightly fitted into the guides. Infusions (0.5 µl/side) were carried out over a 60 s period and the cannulae were left in place for 60 additional seconds to minimize backflow. The placement of the cannulae was verified postmortem: 2-4 h after the last behavioral test, 0.8 µl of a 4% methylene-blue solution was infused as described above and the spread of the dye 30 min thereafter was taken as an indication of the presumable diffusion of the vehicle or drug previously given to each animal. Only data from animals with correct cannulae implants were analyzed. All procedures were conducted in accordance with the ‘Principles of laboratory animal care’ (NIH publication No. 85-23, revised). After recovery from surgery, animals were trained in step-down inhibitory avoidance as described in detail elsewhere^4,27^ and immediately after training^2-5,27^ (Figure 1), or 5 min prior to testing 24 h later (Figure 2), they were infused bilaterally with 0.5 µl of 0.01, 1 or 10 pmoles of OA (Calbiochem) or its vehicle (20% dimetylsulfoxide). This lowest dose of OA is known to selectively inhibit PP1; intermediate doses do not affect the activity of any known phosphatase, while the highest dose of OA selectively inhibits PP2A.^21,29,30^ The infusion cannulae was fitted into the guide, its tip protruded 1 mm beyond that of the guide, and reached the CA1 region. Animals were tested for STM and LTM at 3h and 24h after training, respectively,^27^ and their latency to step-down from the platform onto the floor grid was measured automatically. Upon placing their four paws on the grid they received a 0.4 mA, 2 sec scrambled footshock on the training session. No footshocks were delivered during the STM or LTM test sessions.^3-5,27^ As is customary,^2-5.27,28^ the posttraining infusions were used to study drug effects on STM and/or LTM consolidation, and the pre-test infusions were used to study drug effects on retrieval.

To identify any unspecific side effects of the treatments on locomotor or exploratory activity we examined the effect of the doses of okadaic acid that significantly influenced memory on performance in an open-field task. The animals’ capacity of habituation to the novel environment (a 50 cm high, 50 cm wide and 39 cm deep open-field made of plywood painted white), and their locomotion and rearing was measured during a 5-min session. Locomotion was evaluated by counting crossings of black lines drawn on the floor of the cbox that divided it into 12 equal rectangles. In order to detect habituation performance of crossings and rearings in the first half of the session (2.5 min), these were compared to performances during the second half of the session.^33^ Habituation was measured as a significant decrease in both responses during the two halves of the session.

Only behavioral data from animals with correct cannulae placement was included in the final statistical analysis (Kruskal Wallis test followed by Mann Whitney for comparison among groups), as confirmed by histological control of cannulae placement.

## Results

As shown in Figure 1, the intrahippocampal administration of 0.01 pmol of okadaic acid per side caused full amnesia for both STM and LTM on the inhibitory avoidance task. The 1 pmol dose was not effective and, surprisingly, the 10 pmol/side dose had a positive effect on LTM retention.

In addition, as shown in Figure 2, the two doses of okadaic acid that affected consolidation of the avoidance task had no effect on retrieval when given 5 min prior to the STM or the LTM test.

Table 1 illustrates that none of the treatments affected locomotion or exploration or their habituation in a 5 min open field session.

**Table 1:** Effect of intrahippocampal infusion of okadaic acid at doses that efficiently affected inhibitory avoidance memory (0.01 and 10 pmol/side) on crossings and rearings in the open field. Animals received bilateral infusions of vehicle (20% dimethylsulfoxide in saline) or okadaic acid into the hippocampus bilaterally 15 minutes prior to being placed in the open field. Data are shown as means±standard deviations of total responses in the 5 min session, followed by the number of responses in the first and in the second half of the session.

Group	Rearing responses				Crossings		
	**Total rearings**	**Rearings 0-2.5 min**	**Rearings 2.5-5 min**		**Total crossings**	**Crossings 0-2.5 min**	**Crossings 2.5-5 min**
Vehicle	8.1±3.5	6.0±2.0	2.1±1.7^as^		48.1±31.0	25.3±9.6	12.8±8.6^a^
OA 0.01 pmol/side	12.0±8.5	8.3±6.2	3.7±3.1^a^		55.8±22.9	32.6±14.6	23.2±13.6^a^
OA 10.0 pmol/side	9.5±6.9	6.5±4.3	3.1±3.5^a^		47.3±22.1	34.3±18.4	13.0±11.4^a^

a indicates significant difference between the two halves of the session at p<0.05 level on a post-hoc Duncan test. There was habituation of both crossings and rearings at each 2.5 min block (0-2.5 min and 2.5-5 min) for each group tested. No significant difference was found among all groups, and okadaic acid had no effect on this relationship.

## Discussion

The amnestic effect of OA on both STM and LTM corroborate previous findings of acute and chronic treatments with OA involving various tasks and species.^12,19.21,33^ At a dose known to selectively inhibit PP1 (0.01 pmol/side),^21,29,30^ post-training intrahippocampal OA depressed both STM and LTM. At a dose known to selectively inhibit PP2A but not PP1 (10 pmol/side),^21,29,30^ post-training hippocampal OA specifically enhanced LTM consolidation.^2,28^ At an intermediate dose (1 pmol) which does not inhibit either enzyme,^29,30^ OA had no effect on either of the two forms of memory. Whether administered at the lower or at the higher dose, intrahippocampal OA given prior to retention testing had no effect on retrieval.

PP1 and PP2A interact with, and modulate, several intracellular signaling pathways known to influence LTD, LTP and LTM consolidation.^13-16.22,23^ The present findings provide no clues as to what specific system(s) participate in the amnesic influence of OA at the lower dose, or the mechanisms underlying the enhancing effect on LTM at the highest dose. Nevertheless, the latter phenomenon clearly points to an inhibitory role of endogenous PP2A in LTM consolidation.

Phosphatases, in particular calcineurin and PP1,^25^ have been suggested to act as inhibitory constraints to memory formation^25^ and, alternatively, to represent mechanisms of active forgetting.^34^ Although the evidence available does not allow us to determine which is the most accurate of these descriptions, both hypotheses reinforce the well-known complexity of the cognitive processes and point to phosphatases as important factors. The importance of a degree of forgetting^34,35^ in order to establish new or important memories has been recently studied in detail,^35^ including its implications in terms of catabolic biochemical processes.^36^ It is possible that the enhancement of LTM formation by OA, at the dose that inhibits PP2A, may be related to this forgetting activity.
